# Real-Time Polyp Detection, Localization and Segmentation in Colonoscopy Using Deep Learning

**DOI:** 10.1109/ACCESS.2021.3063716

**Published:** 2021-03-04

**Authors:** Debesh Jha, Sharib Ali, Nikhil Kumar Tomar, Håvard D. Johansen, Dag Johansen, Jens Rittscher, Michael A. Riegler, Pål Halvorsen

**Affiliations:** 1 SimulaMet0167OsloNorway; 2 Department of Engineering ScienceBig Data Institute, University of Oxford 6396 OxfordOX3 7XFU.K.; 3 Department of Computer ScienceUiT–The Arctic University of Norway9037TromsøNorway; 4 Oxford NIHR Biomedical Research CentreOxfordOX4 2PGvU.K.; 5 Department of Computer ScienceOslo Metropolitan University 60499 0167OsloNorway

**Keywords:** Medical image segmentation, ColonSegNet, colonoscopy, polyps, deep learning, detection, localisation, benchmarking, Kvasir-SEG

## Abstract

Computer-aided detection, localisation, and segmentation methods can help improve colonoscopy procedures. Even though many methods have been built to tackle automatic detection and segmentation of polyps, benchmarking of state-of-the-art methods still remains an open problem. This is due to the increasing number of researched computer vision methods that can be applied to polyp datasets. Benchmarking of novel methods can provide a direction to the development of automated polyp detection and segmentation tasks. Furthermore, it ensures that the produced results in the community are reproducible and provide a fair comparison of developed methods. In this paper, we benchmark several recent state-of-the-art methods using Kvasir-SEG, an open-access dataset of colonoscopy images for polyp detection, localisation, and segmentation evaluating both method accuracy and speed. Whilst, most methods in literature have competitive performance over accuracy, we show that the proposed ColonSegNet achieved a better trade-off between an average precision of 0.8000 and mean IoU of 0.8100, and the fastest speed of 180 frames per second for the detection and localisation task. Likewise, the proposed ColonSegNet achieved a competitive dice coefficient of 0.8206 and the best average speed of 182.38 frames per second for the segmentation task. Our comprehensive comparison with various state-of-the-art methods reveals the importance of benchmarking the deep learning methods for automated real-time polyp identification and delineations that can potentially transform current clinical practices and minimise miss-detection rates.

## I. Introduction

Colorectal Cancer (CRC) has the third highest mortality rate among all cancers. The overall five-year survival rate of colon cancer is around 68%, and stomach cancer is only around 44% [1]. Searching for and removing precancerous anomalies is one of the best working methods to avoid CRC based mortality. Among these abnormalities, polyps in the colon are important to detect because it can develop into the CRC at late stage. Thus, an early detection of CRC is crucial for survival.

After modification in the lifestyle, the prevention from the CRC is the screening of the colon regularly. Different research studies suggest that population-wide screening advances the prognosis and can even reduce the incidence of CRC [2]. Colonoscopy is an invasive medical procedure where an endoscopist examines and operates on the colon using a flexible endoscope. It is considered to be the best diagnostic tool for colon examination for early detection and removal of polyps. Therefore, colonoscopic screening is the most preferred technique among gastroenterologists.

Polyps are abnormal growths of tissue protruding from the mucous membrane. They can occur anywhere in the gastrointestinal (GI) tract but are mostly found in the colorectal area and are often considered a predecessor of CRC [3], [4]. Polyps may be pedunculated (having a well-defined stalk) or sessile (without a defined stalk). The colorectal polyps can be categorised into two classes: non-neoplastic and neoplastic. Non-neoplastic polyps are further sub-categorised into hyperplastic, inflammatory, and hamartomatous polyps. These types of polyps are non-cancerous and not harmful. Neoplastic is further sub-categorised into adenomas and serrated polyps. These polyps can develop into the risk of cancer. Based on their size, colorectal polyps can be categorised into three classes, namely, diminutive (≤5mm), small (6 to 9 mm), and advanced (large) (≥10mm) [5]. Usually, larger polyps can be detected and resected.

There exists a significant risk with small and diminutive colorectal polyps [6]. A polypectomy is a technique for the removal of small and diminutive polyps. There are five different polypectomy techniques for resection of diminutive polyps, namely, cold forceps polypectomy, hot forceps polypectomy, cold snare polypectomy, hot snare polypectomy, and endoscopic mucosal resection [5]. Among these techniques, cold snare polypectomy is considered best polypectomy technique for resectioning small colorectal polyps [7].

Colonoscopy is an invasive procedure that requires high-quality bowel preparation as well as air insufflation during examination [8]. It is both an expensive and time-demanding procedure. Nevertheless, on average, 20% of polyps are missed during examinations. The risk of getting cancer therefore relates to the individual endoscopists’ ability to detect polyps [9]. Recent studies have shown that new endoscopic devices and diagnostic tools have improved the adenoma detection rate and polyp detection rate [10], [11]. However, the problem of over-looked polyps remains the same.

The colonoscopy videos recorded at the clinical centers store a significant amount of colonoscopy data. However, the collected data are not used efficiently as they are labour intense for the endoscopists [12]. Thus, a second review of videos are often not done. This might lead to missed detection at an early stage largely. Automated data curation and annotation of video data is a prerequisite for building reliable Computer Aided Diagnosis (CADx) systems that can help to assess clinical endoscopy more thoroughly [13]. A fraction of the collected colonoscopy data can be curated to develop computer-aided systems for automated detection and delineation of polyps either during the clinical procedure or after the reporting. At the same time, to build a robust system, it is vital to incorporate data variability related to patients, endoscopic procedure, and endoscope manufacturers. Even though recent developments in computer vision and system designs have enabled us to built accurate and efficient systems, these largely depend on the data availability as most recent methods are data voracious. The lack of availability of public datasets [14] is a critical bottleneck to accelerate algorithm development in this realm.

In general, curating medical datasets are challenging and it requires domain knowledge expertise. Reaching a consensus to achieve ground truth labels from different experts on the same dataset is again another obstacle. Typically, in colonoscopy, smaller polyps or flat/sessile polyps that are usually missed out during a procedure can be difficult to observe even during manual labeling. Other challenges include the patient variability and presence of different sizes, shapes, textures, colors, and orientations of these polyps [3]. Therefore, during polyp data curation and developing of automated systems for the colonoscopy, it is vital that all various challenges often come along routine colonoscopy has to be taken into consideration.

Automatic polyp detection and segmentation systems based on Deep Learning (DL) have a high overall performance in both colonoscopy images and colonoscopy videos [15], [16]. Ideally, the automatic CADx systems for polyps detection, localisation, and segmentation should have: 1) consistent performance and improved robustness to patient variability, i.e., the system should be able to produce reliable outputs, 2) high overall performance surpassing the set bar for algorithms, 3) real-time performance required for clinical applicability, and 4) easy-to-use system that can provide with clinically interpretable outputs. Scaling this to a population sized cohort is also a very resource-demanding and incurs enormous costs. As a first step, we therefore target the detection, localisation, and segmentation of colorectal polyps known as precursors of CRC. The reason for starting with this scenario is that most colon cancers arise from benign adenomatous polyps (around 20%) containing dysplastic cells. Detection and removal of polyps prevent the development of cancer, and the risk of getting CRC in the following 60 months after a colonoscopy depends largely on the endoscopist ability to detect polyps [9].

Detection and localisation of polyps are usually critical during routine surveillance and to measure the polyp load of the patient at the end of the surveillance while pixel-wise segmentation becomes vital to automate the polyp boundary delineation during the surgical procedures or radio-frequency ablations. In this paper, we evaluate DL methods for both detection (and localisation referring to bounding box detection) and segmentation (pixel-wise classification or semantic segmentation) SOTA methods on Kvasir-SEG dataset [17] to provide a comprehensive benchmark for the colonoscopy images. The main aim of the paper is to establish a new strong benchmark with existing successful computer vision approaches. Our contributions can be summarised as follows:

• We propose ColonSegNet, an encoder-decoder architecture for segmentation of colonoscopic images. The architecture is very efficient in terms of processing speed (i.e., produces segmentation of colonoscopic polyp in real-time) and competitive in terms of performance.
• A comprehensive comparison of the state-of-the-art computer vision baseline methods on the Kvasir-SEG dataset is presented. The best approaches show real-time performance for polyp detection, localisation, and segmentation.
• We have established strong benchmark for detection and localisation on the Kvasir-SEG dataset. Additionally, we have extended segmentation baseline as compared to [3], [17], [18]. These benchmarks can be useful to develop reliable and clinically applicable methods.
• Detection, localisation, and semantic segmentation performances are evaluated on standard computer vision metrics.
• Detailed analysis have been presented with the specific focus on the best and worst performing cases that will allow to dissect method success and failure modes required to accelerate algorithm development.

The rest of the paper is organized as follows: In Section II, we present related work in the field. In Section III, we present the material. Section IV presents both detection, localisation, and segmentation methods. Result are presented in Section V. Discussion on the best performing detection, localisation, and semantic segmentation approaches are presented in Section VI and finally a conclusion is provided in the Section VII.

## II. Related Work

Automated polyp detection has been an active topic for research over the last two decades and considerable work has been done to develop efficient methods and algorithms. Earlier works were especially focused on polyp color and texture, using handcrafted descriptors-based feature learning [27], [28]. More recently, methods based on Convolutional Neural Networks (CNNs) have received significant attention [29], [30], and have been the go to approach for those competing in public challenges [31], [32].

Wang *et al.* [33] designed algorithms and developed software modules for fast polyp edge detection and polyp shot detection, including a polyp alert software system. Shin *et al.* [34] have used region-based CNN for automatic polyp detection in colonoscopy videos and images. They used Inception ResNet as a transfer learning approach and post-processing techniques for reliable polyp detection in colonoscopy. Later on, Shin *et al.* [14] used generative adversarial network [35], where they showed that the generated polyp images are not qualitatively realistic; however, they can help to improve the detection performance. Lee *et al.* [15] used YOLO-v2 [36], [37] for the development of polyp detection and localisation algorithm. The algorithm produced high sensitivity and near real-time performance. Yamada *et al.* [38] developed an artificial intelligence system that can automatically detect the sign of CRC during colonoscopy with high sensitivity and specificity. They claimed that their system could aid endoscopists in real-time detection to avoid abnormalities and enable early disease detection.

In addition to the work related to automatic detection and localisation, pixel-wise classification (segmentation) of the disease provides an exact polyp boundary and hence is also of high significance for clinical surveillance and procedures. Bernel *et al.* [31] presented the results of the automatic polyp detection subchallenge, which was the part of the endoscopic vision challenge at the Medical Image Computing and Computer Assisted Intervention (MICCAI) 2015 conference. This work compared the performance of eight teams and provided an analysis of various detection methods applied on the provided polyp challenge data. Wang *et al.* [16] proposed a DL-based SegNet [39] that had a real-time performance with an inference of more than 25 frames per second. Geo and Matuszewski [40] used fully convolution dilation networks on the Gastrointestinal Image ANAlysis (GIANA) polyp segmentation dataset. Jha *et al.* [3] proposed ResUNet++ demonstrating 10% improvement compared to the widely used UNet baseline on Kvasir-SEG dataset. They also further applied the trained model on the CVC-ClinicDB [23] dataset showing more than 15% improvement over UNet. Ali *et al.* [32] did a comprehensive evaluation for both detection and segmentation approaches for the artifacts present clinical endoscopy including colonoscopy data [41]. Wang *et al.* [42] proposed a boundary-aware neural network (BA-Net) for medical image segmentation. BA-Net is an encoder-decoder network that is capable of capturing the high-level context and preserving the spatial information. Later on, Jha *et al.* [43] proposed DoubleUNet for the segmentation, which was applied to four biomedical imaging datasets. The proposed DoubleUNet is the combination of two UNet stacked on top of each other with some additional blocks. Experimental results on CVC-Clinic and ETIS-Larib polyp datasets show the state-of-the-art (SOTA) performances. In addition to the related work on polyp segmentation, there are studies on segmentation approaches [44]–[47].

Datasets has been instrumental for medical research. Table 1 shows the list of the available endoscopic image and video datasets. Kvasir-SEG, ETIS-Larib, and CVC-ClinicDB contain colonoscopy images, whereas Kvasir, Nerthus, and HyperKvasir contain the images from the whole GI. KvasirCapusle contains images from video capsule endoscopy. All the dataset contains images acquired from conventional White Light (WL) imaging technique except the EDD dataset, where it contains images from both WL imaging and Narrow Band Imaging (NBI) techniques. All of these datasets contain at least a polyp class. Out of nine available datasets, Kvasir-SEG [17], ETIS-Larib [22], and CVC-ClinicDB [23] has manually labeled ground truth masks. Among them, Kvasir-SEG offers the most number of annotated samples providing both ground truth masks and bounding boxes offering detection, localisation, and segmentation task. All of the datasets are publicly available.

**TABLE 1:** Available Endoscopic Datasets

Dataset	Organ	Source	Findings	Dataset content	Task type
Kvasir-SEG [17]	Large bowel	WL^⋄^	Polyp	1000 images	Detection, localisation & segmentation
Kvasir [19]	Whole GI	WL^⋄^	Polyps, esophagitis, ulcerative colitis, z-line, pylorus, cecum, dyed polyp, dyed resection margins, stool	8,000 images	Classification
Nerthus [20]	Large bowel	WL^⋄^	Stool - categorization of bowel cleanliness	21 videos	Classification
HyperKvasir [21]	Whole GI	WL^⋄^	16 different classes from upper GI & 24 different classes from lower GI tract	110,079 images & 373 videos	Classification
ETIS-Larib [22]	Colonoscopy	WL^⋄^	Polyp	196 images	Segmentation
CVC-Clinic [23]	Colonoscopy	WL^⋄^	Polyp	612 images	Segmentation
KvasirCapsule [24]	Whole GI	VCE	13 different classes of GI anomalies	4,820,739 images & 118 videos	Classification
EDD 2020 [25]	Entire GI	NBI^†^, WL^⋄^	Polyp, Barrett’s esophagus, high-grade dysplasia, suspicious (low-grade), cancer	386 images	Detection, localisation & segmentation
Kvasir-Instrument [17]	Large Bowel	WL^⋄^	Tools and instruments	590 images	Detection, localization, Segmentation

^†^ Narrow band imaging

^⋄^ White light imaging

Dataset development, benchmarking of the methods, and evaluation are critical in the medical imaging domain. It inspires the community to build clinically transferable methods on a well-curated and standardised dataset. Due to the lack of benchmark papers, it becomes utmost difficult to understand the clear strength of methods in the literature. New algorithm developments demonstrating its translational abilities in clinics is thus very minimal. Data science challenges do offer some insight, however, a comprehensive analysis on various different aspects such as detection, localisation, segmentation, and inference time estimation are still not covered by the most.

Inspired by the previous benchmark for polyp detection [31], endoscopic artifact detection [41], endoscopic disease detection and segmentation [25], endoluminal scene object segmentation [48], and endoscopic instrument segmentation [49], we introduce a new benchmark for the automatic polyp detection, localisation and segmentation using publicly available Kvasir-SEG dataset.

## III. Materials – Dataset

**FIGURE 1.**
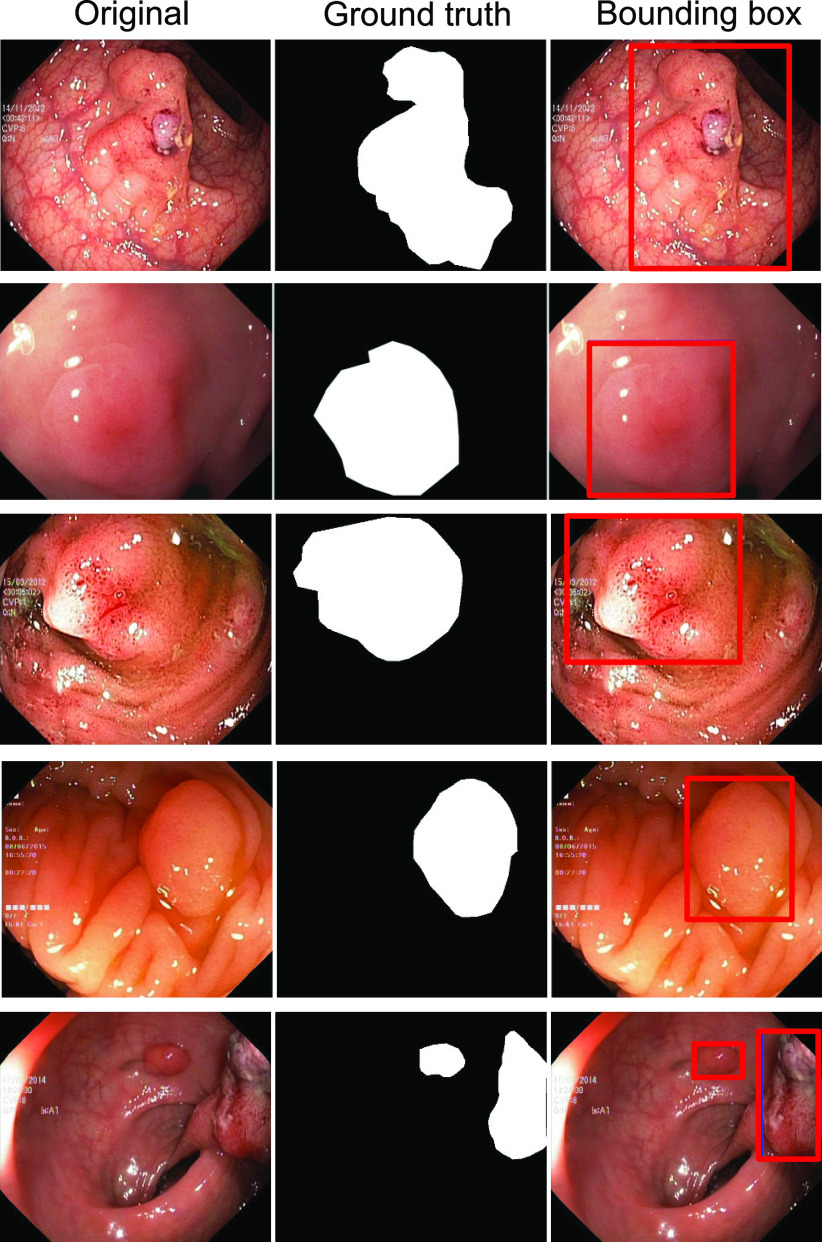
Sample images from Kvasir-SEG dataset: Annotated masks (2nd column) and bounding boxes (3rd column) for selected samples.

We have used the Kvasir-SEG [17] for detection, localisation, and segmentation tasks. Figure 1 shows the image, ground truth information, and their detection (their localised bounding boxes in red). This dataset is the outcome of an initiative for open and reproducible results. It contains 1000 polyp images acquired by high-resolution electromagnetic imaging system, i.e., ScopeGuide, Olympus Europe, their corresponding masks and bounding box information. The images and their ground truths can be used for the segmentation task, whereas the bounding box information provides an opportunity for the detection task. The resolution of the images in this dataset ranges from $332\times 487$ to $1920\times 1072$ pixels. The dataset can be downloaded at https://datasets.simula.no/kvasir-seg/. The dataset includes images of 700 large polyps ($> 160 \times 160$ pixels), 323 medium sized polyps (> $64\times 64$ pixels and $\leq 160 \times 160$ pixels) and 48 small polyps ($\leq 64\times 64$ pixels). In total, the dataset consists of 1072 images of polyps with segmentation masks and bounding boxes.

## IV. Method

Detection methods aim to predict the object class and regress bounding boxes for localisation, while segmentation methods aim to classify the object class for each pixel in an image. In Figure 1, ground truth masks for segmentation task are shown in 2nd column while corresponding bounding boxes for the detection task are in 3rd column. This section describes the baseline methods for detection, localisation and segmentation methods used for the automated detection and segmentation of polyp in the Kvasir-SEG dataset.

### A. Detection and Localisation Baseline Methods

**FIGURE 2.**
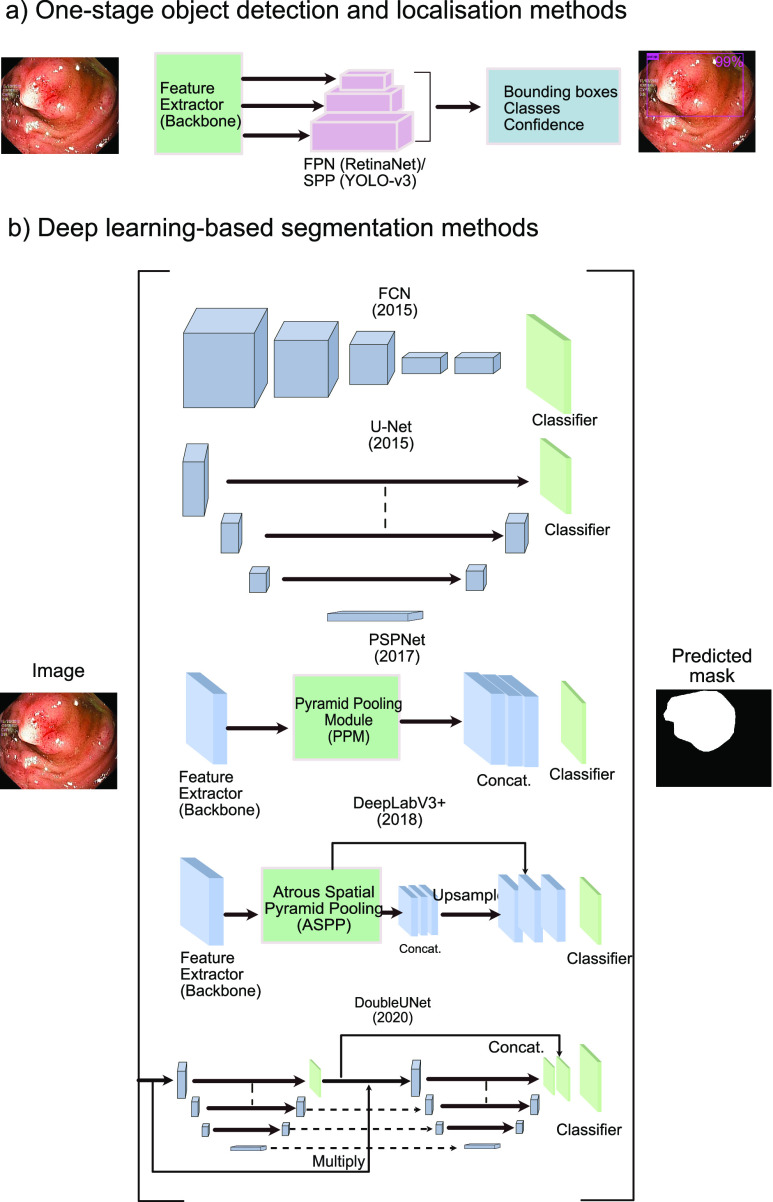
Baseline detection, localisation and semantic segmentation method summary.

Detection methods consist of input, backbone, neck, and head. The input can be images, patches, or image pyramids. The backbone can be different CNN architectures such as VGG16, ResNet50, ResNext-101, and Darknet. The neck is the subset of the backbone network, which could consist of FPN, PANet, and Bi-FPN. The head is used to handle the prediction boxes that can be one stage detector for dense prediction (e.g., YOLO, RPN, and RetinaNet [50]), and two-stage detector with the sparse prediction (e.g., Faster R-CNN [51] and RFCN [52]). Recently, one stage methods have attracted much attention due to their speed and ability to obtain optima accuracy. This has been possible because recent networks utilise feature pyramid networks or spatial-pyramid pooling layers to predict candidate bounding boxes which are regressed by optimising loss functions (see Figure 2).

In this paper, we use EfficientDet [53] which uses EfficientNet [54], as the backbone architecture, bi-directional feature pyramid network (BiFPN) as the feature network, and shared class/box prediction network. Additionally, we also use Faster R-CNN [51], which uses region proposal network (RPN), as the proposal network and Fast R-CNN [55] as the detector network. Moreover, we use YOLOv3 [56] that utilises multi-class logistic loss (*binary cross-entropy* for classification loss and *mean square error* for regression loss) modeled with regularizers such as objectness prediction scores. Furthermore, we also used YOLOv4 [57], which utilises an additional bounding box regressor based on the Intersection over Union (IoU) and a cross-stage partial connections in their backbone architecture. Additionally, YOLOv4 allows on fly data augmentation, such as mosaic and cut-mix.

RetinaNet [50] takes into account the data driven property that allows the network to focus on “hard” samples for improved accuracy. The easy to adapt backbones for feature extraction at the beginning of the network provides the opportunity to experiment with deeper and varied architectures such as ResNet50, and ResNet101 for RetinaNet and 53 layered Darknet53 backbone for YOLOv3 and YOLOv4 architecture. To tackle the different aspect ratio problem, for both one stage networks, optimal anchor boxes [51] are searched and pre-defined for the provided data to tackle large variance of scale and aspect ratio of boxes. Table 2 shows the hyperparameter used by each of the object detection methods for the detection task.

**TABLE 2:** Hyperparameters Used for Baseline Methods for polyp Detection and Localisation Task on Kvasir-SEG. Here, CIoU: Complete Intersection-of-Union Loss, MSE: Mean Square Error, CE: Cross-Entropy

Method	Learning rate	Optimizer	Batch size	Loss	Anchors	Threshold
Faster R-CNN [50]	2.5e^−4^	Adam	8	$\text{L}1^{smooth}$, log-loss	256	0.4
RetinaNet [49]	1e^−5^	SGD	8	$\text{L}1^{smooth}$, focal loss	15 (pyramid)	0.3
YOLOv3+spp [55]	1e^−3^	SGD	16	MSE, CE	8	0.25
YOLOv4 [56]	1e^−3^	SGD	16	CIoU, CE	8	0.25
EfficientDet-D0 [52]	1e^−4^	Adam	8	Focal loss	default	0.4

**TABLE 3:** Result on the polyp Detection and Localisation Task on the Kvasir-SEG Dataset. Two Best Scores are Highlighted in Bold

Method	Backbone	AP	IoU	AP_25_	AP_50_	AP_75_	FPS
EfficientDet-D0 [52]	EfficientNet-b0, biFPN	0.4756	0.4322	0.6846	0.5047	0.2280	35.00
Faster R-CNN [50]	ResNet50	0.7866	0.5621	0.8947	0.8418	0.5660	8.0
RetinaNet [49]	ResNet50	0.8697	0.7313	0.9395	0.9095	0.6967	16.2
RetinaNet [49]	ResNet101	0.8745	0.7579	0.9483	0.9095	0.7132	16.8
YOLOv3+spp [55]	Darknet53	0.8105	0.8248	0.8856	0.8532	0.7586	45.01
YOLOv4 [56]	Darknet53, CSP	0.8513	0.8025	0.9123	0.8234	0.7594	48.00
ColonSegNet (Proposed)	–	0.8000	0.8100	0.9000	0.8166	0.6706	180.00

### B. Segmentation Baseline Methods

In the past years, data-driven approaches using CNNs have changed the paradigm of computer vision methods, including segmentation. An input image can be directly be fed to convolution layers to obtain feature maps, which can be later upsampled to predict pixel-wise classification providing object segmentation. Such networks learn from available ground truth labels and can be used to predict labels from other similar data. A Fully Convolutional Network (FCN) based segmentation was first proposed by Long *et al.* [58] that can be trained end-to-end. Ronneberger *et al.* [59] modified and extended the FCN architecture to a UNet architecture. The UNet consist of an analysis (*encoder*) and a synthesis (*decoder*) path. In the analysis path of the network, deep features are learnt, whereas in the synthesis path segmentation is performed on the basis of the learnt features.

Pyramid Scene Parsing Network (PSPNet) [60] introduced a pyramid pooling module aimed at aggregating global context information from different regions which are upsampled and concatenated to form the final feature representation. A final per-pixel prediction is obtained after a convolution layer (see Figure 2, third architecture). For feature extraction, we have used the ResNet50 architecture pretrained on imageNet. Similar to the UNet architecture, DeepLabV3+ [61] is an encoder-decoder network. However, it utilizes atrous separable convolutions and spatial pyramid pooling (see Figure 2, last architecture) for fast inference and improved accuracy. Atrous convolution controls the resolution of features computed and adjust the receptive field to effectively capture multi-scale information. In this paper, we have used an output stride of 16 for both encoder and decoder networks of DeepLabV3+ and have experimented on both ResNet50 and ResNet101 backbones.

ResUNet [62] integrates the power of both UNet and residual neural network. ResUNet++ [3] is the improved version of ResUNet architecture. It has additional layers including squeeze-and-excite block, Atrous Spatial Pyramid Pooling (ASPP), and attention block. These additional layers helps learning the deep features that are capable of improved prediction of pixels for object segmentation tasks. DoubleU-Net [43] consists of two modified UNet architecture. It uses VGG-19 pretrained on ImageNet [63] as the first encoder. The main reason behind using VGG-19 (similar to UNet [64]) was that it is a lightweight model. The additional component in the DoubleUNet are squeeze-and-excite block, and ASPP block. High-Resolution Network (HRNet) [65] maintains high-resolution representation convolution in parallel and interchange the information across the resolution continuously. This is one of the most recent and popular method in the literature. Furthermore, we have used UNet with ResNet34 as a backbone network and trained the model to compare with the other state-of-the-art semantic segmentation networks.

Table 4 shows the hyperparameters used for each of the semantic segmentation based benchmark methods used. From the table, we can see that number of trainable parameters of the baseline methods are large. A high number of trainable parameters in the network makes it complex, leading to a lower frame rate. It is therefore essential to design an efficient, lightweight architecture that can provide a higher frame rate and better performance. In this regard, we propose a novel architecture, ColonSegNet, that requires only few number of training parameters, which can save training and inference time. More details about the architecture can be found in the below section.

**TABLE 4:** Hyperparameters Used for Baseline Methods for polyp Segmentation Task on Kvasir-SEG Dataset

Method	No. of parameters	Learning rate	Optimizer	Batch size	Loss	Momentum	Decay rate
UNet [58]	7,858,433	1e^−2^	SGD	8	Cross-entropy	–	–
ResUNet [61]	8,420,077	1e^−4^	Adam	8	Dice loss	–	–
ResUNet++ [3]	16,242,785	1e^−4^	Adam	8	Dice loss	–	–
HRNet [64]	9,524,036	1e^−4^	Adam	8	Dice loss	–	–
DoubleUNet [42]	29,303,426	1e^−4^	Adam	8	Dice loss	–	–
PSPNet [59]	48,631,850	1e^−2^	SGD	8	Cross-entropy	–	–
DeepLabv3+ [60]	ResNet50: 39,756,962	1e^−2^	SGD	8	Cross-entropy	0.9	1e^−4^
DeepLabv3+ [60]	ResNet101: 58,749,090	1e^−3^	SGD	8	Cross-entropy	0.9	1e^−4^
FCN8 [57]	134,270,278	1e^−2^	SGD	8	Cross-entropy	0.9	1e^−4^
UNet-ResNet34	33,509,098	1e^−5^	Adam	8	Cross-entropy	0.9	1e^−4^
ColonSegNet (Proposed)	5,014,049	1e-4	Adam	8	Cross-entropy + Dice loss	–	–

### C. ColonSegNet

**FIGURE 3.**
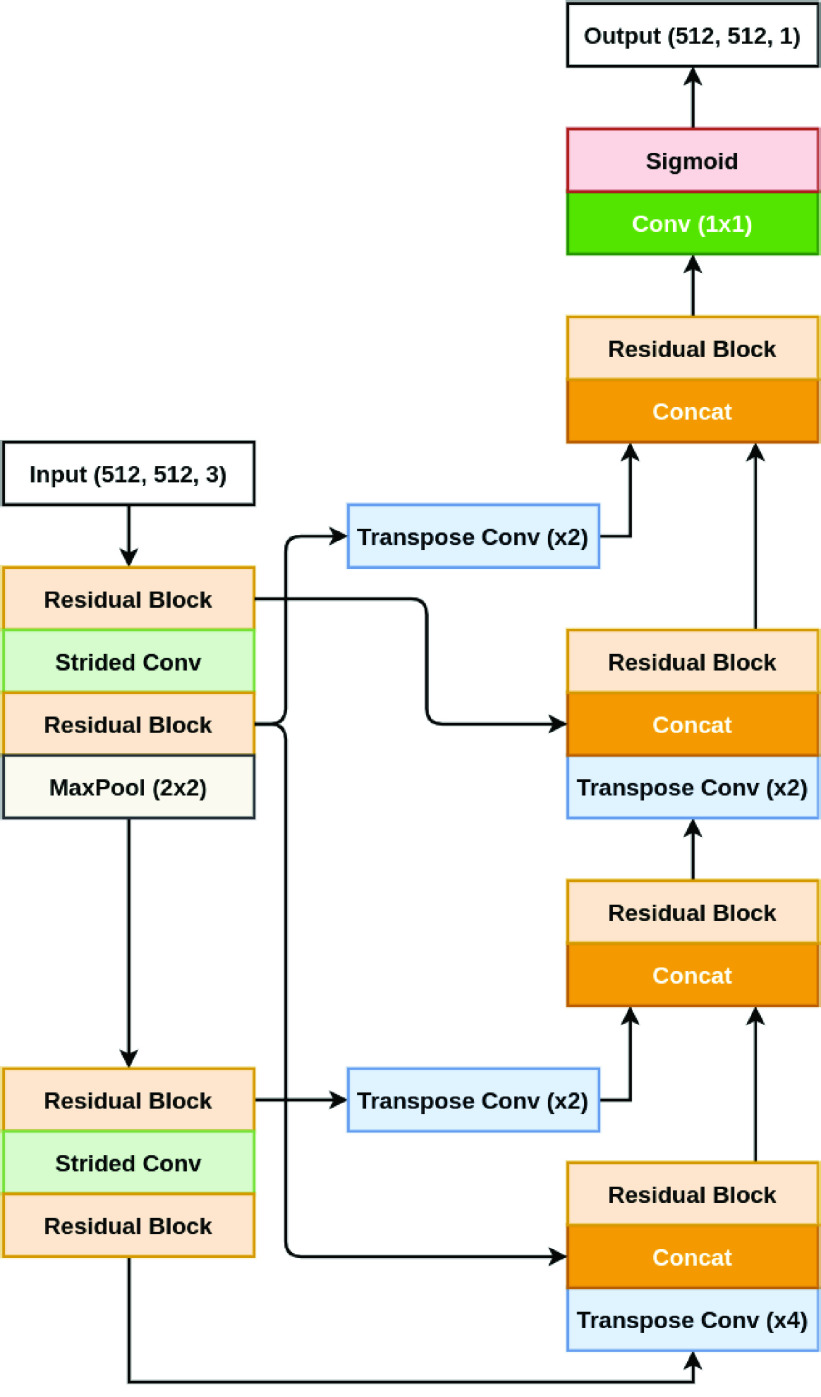
Block diagram of ColonSegNet.

Figure 3 shows the block diagram of the proposed ColonSegNet. It is an encoder-decoder that uses residual block [66] with squeeze and excitation network [67] as the main component. The network is designed to have very few trainable parameters as compared to other networks baseline networks such as U-Net [59], PSPNet [60], DeepLabV3+ [61], and others. The use of fewer trainable parameters makes the proposed architecture a very light-weight network that leads to real-time performance.

The network consists of two encoder blocks and two decoder blocks. The encoder network learns to extract all the necessary information from the input image, which is then passed to the decoder. Each decoder block consists of two skip connections from the encoder. The first is a simple concatenation, and the second skip connection passed through a transpose convolution to incorporates multi-scale features in the decoder. These multi-scale features help the decoder to generate more semantic and meaningful information in the form of a segmentation mask.

The input image is fed to the first encoder, which consists of two residual blocks and a $3 \times 3$ strided convolution in between them. This layer is followed by a $2 \times 2$ max-pooling. Here, the output feature map spatial dimensions are reduced to $\frac {1}{4}$ of the input image. The second encoder consists of two residual blocks and a $3 \times 3$ strided convolution in between them.

The decoder starts with a transpose convolution, where the first decoder uses a stride value 4, which increases the feature map spatial dimensions by 4. Similarly, the second decoder uses a stride value of 2, increasing the spatial dimensions by 2. Then, the network follows a simple concatenation and a residual block. Next, it is concatenated with the second skip connection and again followed by a residual block. The output of the last decoder block passes through a $1 \times 1$ convolution and a sigmoid activation function, generating the binary segmentation mask.

#### 1) Data Augmentation

Supervised learning methods are data voracious and require large amount of data to obtain reliable and well-performing models. Acquiring such training data through data collection, curation, and annotation is a manual process that needs significant resources and man-hours from both clinical experts and computational scientists.

Data augmentation is a common technique to computationally increase the number of training samples in a dataset. For our DL models, we use basic augmentation techniques such as horizontal flipping, vertical flipping, random rotation, random scale, and random cropping. The images used in all the experiments undergo normalization and are resized to a fixed size of $512\times 512$. For the normalization, we subtract the image by mean and divide it by standard deviation.

## V. Results

In this section, we first present our evaluation metrics and experimental setup. Then, we present both quantitative and qualitative results.

### A. Evaluation Metrics

We have used standard computer vision metrics to evaluate polyp detection and localisation, and semantic segmentation methods on the Kvasir-SEG dataset.

#### 1) Detection and Localisation Task

For the object detection and localisation task, the commonly used Average Precision (AP) and IoU have been used [68], [69].

• IoU: This metric measures the overlap between two bounding boxes A and B as the ratio between the overlapped area.
  \begin{equation*} \text {IoU(A,B)} =\frac {A \cap B} {A \cup B}\tag{1}\end{equation*}
• AP: AP is computed as the Area Under Curve (AUC) of the precision-recall curve of detection sampled at all unique recall values (r1, r2,…) whenever the maximum precision value drops:
  \begin{equation*} \mathrm {AP} = \sum _{n}{\left \{{\left ({r_{n+1}-r_{n}}\right)p_{\mathrm {interp}}(r_{n+1})}\right \}},\tag{2}\end{equation*}
  with $p_{\mathrm {interp}}(r_{n+1}) =\max \limits_{\tilde {r}\ge r_{n+1}}p(\tilde {r})$. Here, $p(r_{n})$ denotes the precision value at a given recall value. This definition ensures monotonically decreasing precision. AP was computed as an average APs for IoU from 0.25 to 0.75 with a step-size of 0.05 which means an average over 11 IoU levels are used (AP @[.25:.05:.75]).

#### 2) Segmentation Task

For polyp segmentation task, we have used widely accepted computer vision metrics that include Dice Coefficient (DSC), Jaccard Coefficient (JC), precision *(p)*, and recall *(r)*, and overall accuracy *(Acc)*. JC is also termed as IoU. We have also included Frame Per Second (FPS) to evaluate the clinical applicability of the segmentation methods in terms of inference time during the test.

To define each metric, let *tp*, *fp*, *tn*, and fn represents true positives, false positives, true negatives, and false negatives, respectively.

\begin{align*}{ } \text {DSC}=&\frac {2 \cdot tp} {2 \cdot tp + fp + fn} \tag{3}\\ \text {IoU}=&\frac {tp} {tp + fp + fn} \tag{4}\\ { } {r}=&\frac {tp} {tp + fn} \tag{5}\\ { } {p}=&\frac {tp} {tp + fp} \tag{6}\\ { } \text {F2}=&\frac {5p \times r} {4p + r} \tag{7}\\ { } \textit {Acc}=&{\frac {tp + tn} {tp + tn + fp + fn}} \tag{8}\\ { } \text {FPS}=&{\frac {\#frames} {sec}} = {\frac {1} {sec /frame}}\tag{9}\end{align*}

### B. Experimental Setup and Configuration

The methods such as UNet, ResUNet, ResUNet++, DoubleUNet, and HRNet were implemented using Keras [70] with a Tensorflow [71] back-end and were run on a Volta 100 GPU and an Nvidia DGX-2 AI system. A PyTorch implementation for FCN8, PSPNet, DeepLabv3+, UNet-ResNet34, and ColonSegNet networks were done. Training of these methods were conducted on NVIDIA Quadro RTX 6000. NVIDIA GTX2080Ti was used for test inference for all methods reported in the paper. All of the detection methods were implemented using PyTorch and used NVIDIA Quadro RTX 6000 hardware for training the network.

In all of the cases, we used 880 images for training and the remaining 120 images for the validation. Due to different image sizes in the dataset, we resized the images to $512\times 512$. Hyperparamters are important for the DL algorithms to find the optimal solution. However, picking the optimal hyperparameter is difficult. There are algorithms such as grid search, random search, and advanced solutions such as Bayesian optimization for finding the optimal parameters. However, an algorithm such as Bayesian optimization is computationally costly, making it difficult to test several DL algorithms. We have done an extensive hyperparameter search for finding the optimal hyperparameters for polyp detection, localisation, and segmentation task. These sets of hyperparameters were chosen based on empirical evaluation. The used hyperparameters are for the Kvasir-SEG dataset and are reported in the Table 2, and Table 4.

### C. Quantitative Evaluation

#### 1) Detection and Localisation

Table 3 shows the detailed result for the polyp detection and localisation task on the Kvasir-SEG dataset. It can be observed that RetinaNet shows improvement over YOLOv3 and YOLOv4 for mean average precision computed for multiple IoU thresholds and for average precision at IoU threshold 25 (AP_25_) and 50 (AP_50_). RetinaNet with ResNet101 backbone achieved an average precision of 0.8745, while YOLOv4 yielded 0.8513. However, for the IoU threshold of 0.75, YOLOv4 showed improvement over RetinaNet with (AP_75_) of 0.7594 against 0.7132 for RetinaNet with ResNet101 backbone. Similarly, the average IoU of 0.8248 was observed for YOLOv3, which is nearly 8% improvement over RetinaNet. IoU determines the preciseness of the bounding box localisation. EfficientDet-D0 obtained the least AP of 0.4756 and IoU of 0.4322. Faster R-CNN obtained an AP of 0.7866. However, it only obtained an FPS of 8. YOLOv4 with Darknet53 as backbone obtained a FPS of 48, which is $6\times $ faster than Faster R-CNN. The other competitive network was YOLOv3, with an average FPS of 45.01. However, its average precision value is 5% less than YOLOv4. Thus, the quantitative results show that the YOLOv4 with Darknet can detect different types of polyps at a real-time speed of 48 FPS and average precision of 0.8513. Therefore, from the evaluation metrics comparison, YOLOv4 with Darknet53 is the best model for detection and localisation of polyp. The results suggest that the model can help gastroenterologists find missed polyps and decrease the polyp miss-rate. Even though, the proposed ColonSegNet is primary built for real-time segmentation of polyps, we compared the bounding box predictions of the proposed network with SOTA detection methods. It can be observed that the inference of the proposed method is nearly four times faster (180 FPS) than YOLOv4. Additionally, it is also obtaining competitive scores on both AP and IoU metrics (IoU of 0.81 and AP of 0.80). Therefore, it can also be considered as one of the best detection and localisation techniques.

#### 2) Segmentation

Table 5 shows the obtained results on the polyp segmentation task. It can be observed that the UNet with ResNet34 backbone performs better than the other SOTA segmentation methods in terms of DSC, and IoU. However, the proposed ColonSegNet outperforms in terms of processing speed. ColonSegNet is faster than UNet-ResNet34 by more than four times in processing colonoscopy frames. The complexity of the network is six times smaller than the UNet-ResNet34 network. The proposed network is even smaller than the conventional UNet, with its size only being around 0.75 times that of the UNet with higher scores on evaluation metrics compared to the classical UNet and its derivates such as ResUNet and ResUNet++. Additionally, the recall and overall accuracy metrics of ColonSegNet are close to the highest performing UNet-ResNet34 network, which shows the proposed method’s efficiency.

**TABLE 5:** Baseline Methods for polyp Segmentation on the Kvasir-SEG Dataset. Two Best Scores are Highlighted in Bold. “−” Shows That There is no Backbone Used in the Network

Method	Backbone	Jaccard C.	DSC	F2-score	Precision	Recall	Overall Acc.	FPS
UNet [58]	–	0.4713	0.5969	0.5980	0.6722	0.6171	0.8936	11.0161
ResUNet [61]	–	0.5721	0.6902	0.6986	0.7454	0.7248	0.9169	14.8204
ResUNet++ [3]	–	0.6126	0.7143	0.7198	0.7836	0.7419	0.9172	7.0193
FCN8 [57]	VGG 16	0.7365	0.8310	0.8248	0.8817	0.8346	0.9524	24.9100
HRNet [64]	–	0.7592	0.8446	0.8467	0.8778	0.8588	0.9524	11.6970
DoubleUNet [42]	VGG 19	0.7332	0.8129	0.8207	0.8611	0.8402	0.9489	7.4687
PSPNet [59]	ResNet50	0.7444	0.8406	0.8314	0.8901	0.8357	0.9525	16.8000
DeepLabv3+ [60]	ResNet50	0.7759	0.8572	0.8545	0.8907	0.8616	0.9614	27.9000
DeepLabv3+ [60]	ResNet101	0.7862	0.8643	0.8570	0.9064	0.8592	0.9608	16.7500
UNet [58]	ResNet34	0.8100	0.8757	0.8622	0.9435	0.8597	0.9681	35.0000
ColonSegNet (Proposed)	–	0.7239	0.8206	0.8206	0.8435	0.8496	0.9493	182.3812

The original implementation of UNet obtained the least DSC of 0.5969, whereas the UNet with ResNet34 as the backbone model obtained the highest DSC of 0.8757. The second and third best DSC scores of 0.8643 and 0.8572 were obtained for DeepLabv3+ with ResNet101 and DeepLabv3+ with ResNet50 as the backbone, respectively. From the table, it is seen that DeepLabv3+ with ResNet101 performs better than Deeplabv3+ with ResNet50. This may be because of the top-5 accuracy (i.e., the validation results on the ImageNet model) of ResNet101 is slightly better than ResNet50.^1^ Despite of DeepLabv3+ with ResNet101 backbone having the total number of trainable parameters more than 11 times and DeepLabv3+ with ResNet34 being nearly eight times computational complexity, the DSC of ColonSegNet is competitive compared to both of these networks. However in terms, of processing speed, it is almost 11 times faster than DeepLabv3+ with ResNet101 and nearly seven times faster than DeepLabv3 with ResNet34 backbone.^1^https://keras.io/api/applications/

FCN8, HRNet and DoubleUNet provided similar results with DSC of 0.8310, 0.8446, and 0.8129 while ResUNet++ achieved DSC of only 0.7143. A similar trend can be observed for F2-score for all methods. For precision, UNet with ResNet34 backbone achieved the maximum score of $p = 0.9435$, and DeepLabv3+ with ResNet50 backbone achieved the highest scores of $r = 0.8616$, while UNet scored the worst with $p = 0.6722$ and $r = 0.6171$. The overall accuracy was outstanding for most methods, with the highest for UNet and ResNet34 as the backbone. IoU is also provided in the table for each segmentation method for scientific completion. Again, UNet and ResNet34 surpassed others with a mIoU score of 0.8100. Also, UNet and ResNet34 achieved the highest FPS rate of 35 fps, which is acceptable in terms of speed and is relatively faster as compared to DeepLabv3+ with ResNet50 (27.9000) and DeepLabv3+ with ResNet101 (16.7500) and other SOTA methods. Additionally, when we consider the number of parameter uses (see Table 4), UNet with ResNet34 backbone uses less number of the parameters as compared to that of FCN8 or DeepLabv3+ network. Due to the low number of trainable parameters and fastest inference time, ColonSegNet is computationally efficient and becomes the best choice while considering the need for real-time segmentation (182.38 FPS on NVIDIA GTX2080Ti) of polyps with deployment possible on even low-end hardware devices making it feasible for many clinical settings. Whereas, UNet with ResNet34 backbone seems the best choice while taking DSC metric into account, however, with speed of only 35 FPS on NVIDIA GTX2080Ti.

### D. Qualitative Evaluation

**FIGURE 4.**
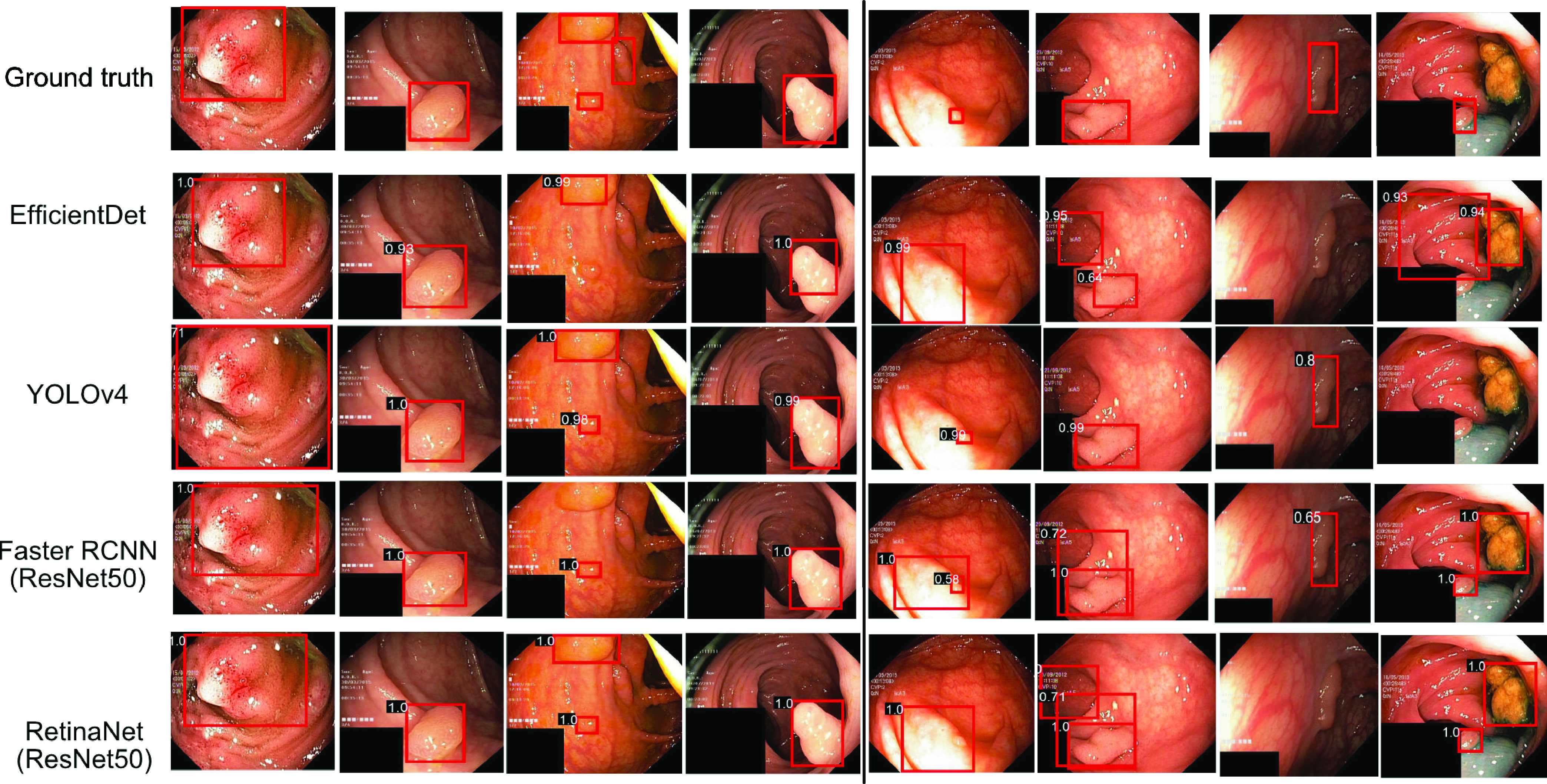
**Detection and localisation results on test dataset:** On right of the black solid line, images where EfficientDet-D0, YOLOv4, Faster R-CNN and RetinaNet (with ResNet50 backbone) have similar results and in most cases obtained highest IoU. On left, images with failed case (worse localisation) for either of the method. Confidence scores are provided on the top-left of the red prediction boxes.

Figure 4 shows the qualitative result for the polyp detection and localisation task along with their corresponding confidence scores. It can be observed that for most images on the left side of the vertical line, both YOLOv4 and RetinaNet are able to detect and localise polyps with higher confidence, except for the third column sample where most of these methods can identify only some polyp areas. Similarly, on the right side of the vertical line, the detected bounding boxes for 5th and 6th column images are too wide for the RetinaNet, while YOLOv4 has the best localisation of polyp (observe the bounding box). Also, in the seventh column, RetinaNet and EfficientDet D0 misses the polyp. In the eighth column, YOLOv4 and EfficientDet D0 misses the small polyp completely while stool and polyp is detected as polyp by the Faster R-CNN and RetinaNet.

**FIGURE 5.**
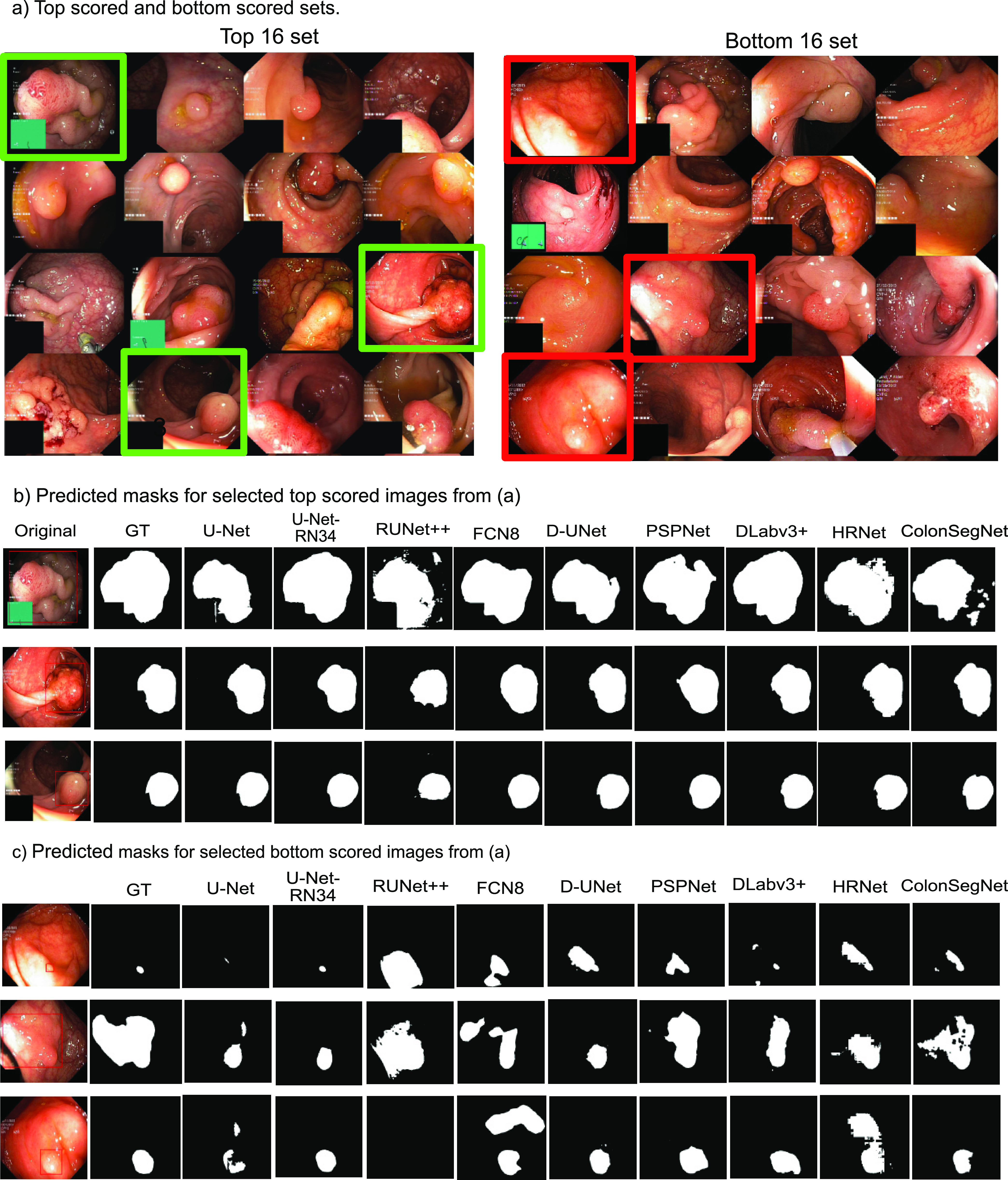
**Best and worse performing samples for polyp segmentation**: a) Top (left) and bottom (right) scored sets, b) predicted masks for top scored images and c) bottom scored images for all methods compared to the ground truth (GT) masks. Green rectangles represent the selected images from top scored set and red rectangle represent those from bottom set. Here, UNet-RN34: UNet-ResNet34, RUNet++: ResUNet++, D-UNet: Double UNet, DLabv3+: DeepLabv3+ (ResNet50).

Figure 5 shows the result for the top-scored and bottom scored sets selected based on their dice similarity coefficient values for the semantic segmentation methods. It can be seen that all the algorithms are able to detect large polyps and produce high-quality masks (see Figure 5(b).

Here, the best obtained segmentation results can be observed for DeepLabv3+ and UNet-ResNet34. However, as shown in Figure 5(c), the segmentation results are affected for flat polyps (very small), images with a certain degree of inclined view, and for the images with saturated areas. The proposed ColonSegNet is able to achieve similar shapes compared to these of the ground truth with some outliers for the predictions which can be seen in Figure 5(b), while for the prediction on worse performing images in Figure 5(c), our proposed network provides comparatively improved predictions on almost all samples.

## VI. Discussion

It is evident that there is a growing interest in the investigation of computational support systems for decision making through endoscopic images. For the first time, we are using Kvasir-SEG for detection and localisation tasks, and comparing segmentation methods with most recent SOTA methods. We provide a reproducible benchmarking of the DL methods using standard computer vision metrics in object detection and localisation, and semantic segmentation. The choice of methods are based their popularity in the medical image domain for detection and segmentation (e.g., UNet, Faster R-CNN), speed (e.g., UNet with ResNet34, YOLOv3), and accuracy (e.g., PSPNet, FCN8, or DoubleUNet) or a combination of all (e.g., DeepLabv3+, YOLOv4).

From the experimental results in Table 3, we can observe that the combination of YOLOv3 with Darknet53 backbone shows improvement over other methods in terms of mIoU, which means a better localisation compared to counterpart RetinaNet. However, YOLOv4 is $3\times $ faster than RetinaNet and has a good trade-off between the average precision and IoU. This is because of their Cross-Stage-Partial-Connections (CSP) and CIoU loss for bounding box regression. However, RetinaNet with the backbone ResNet101 shows competitive results surpassing other methods on average precision but nearly 5% less IoU compared to YOLOv4 and nearly 5% less than YOLOv3-spp. Similarly, state-of-the-art methods Faster R-CNN and EffecientDet-D0 provided the least AP and IoU.

A choice between computational speed, accuracy and precision is vital in object detection and localisation tasks, especially for colonoscopy video data where speed is a vital element to achieve real-time performance. Therefore, we consider YOLOv4 with Darknet53 and CSP backbone as the best approach in the table for the polyp detection and localisation task.

For the semantic segmentation tasks, ColonSegNet showed improvement over all the methods. The method obtained the highest FPS of 182.38. The quantitative results in Figure 5 (b) showed the most accurate delineation of polyp pixels compared to other SOTA methods considered in this paper. The most competitive method to ColonSegNet was UNet with ResNet34 backbone. The other comparable method was DeepLabv3+, which accuracy can be due to its ability to navigate the semantically meaningful regions with its atrous convolution and spatial-pyramid pooling mechanism. Additionally, the feature concatenation from previous feature maps may have helped to compute more accurate maps for object semantic representation and hence segmentation. The other competitor was PSPNet, which is also based on similar idea but on aggregating the global context information from different regions rather than the use of dilated convolutions. The computational speed for DeepLabv3+ with the same ResNet50 backbone as used in PSPNet in our experiments comes from the fact that the 1D separable convolutions and SPP network is used in DeepLabv3+. We evaluated the most recent popular SOTA method in segmentation “HRNet” [65]. While HRNet produced competitive results compared to other SOTA methods, UNet with ResNet34 backbone and DeepLabv3+ outperformed for most evaluation metrics with ColonSegNet being competitive in the recall, and overall accuracy and outperforming other SOTA method significantly.

Figure 5 shows an example for the 16 top scored and 16 bottom scored images on DSC for segmentation. From the results in Figure 5(c), it can be observed that there are polyps whose appearance under the given lighting conditions is very similar to healthy surrounding gastrointestinal skin texture. We suggest that including more samples with variable texture, different lighting conditions, and different angular views (refer to the samples in Figure 5(a) on the right, and (c)) can help to improve the DSC and other metrics of segmentation. We also observed that the presence of sessile or flat polyps were major limiting factors for algorithm robustness. Thus, including smaller polyps with respect to image size can help algorithm to generalise better thereby making these methods more usable for early detection of hard-to find polyps. In this regard, we also suggest the use of spatial pyramid layers to handle small polyps and using context-aware methods such as incorporation of artifacts or shape information to improve the robustness of these methods.

The possible limitation of the study is its retrospective design. Clinical studies are required for the validation of the approach in a real-world setting [72]. Additionally, in the presented study design we have resized the images, which can lead to loss of information and affect the algorithm performance. Moreover, we have optimized all the algorithms based on the empirical evaluation. Even though, optimal hyper-parameters have been set after experiments, we acknowledge that these can be further adjusted. Similarly, meta-learning approaches can be exploited to optimize the hyper-parameters that can work even in resource constraint settings.

## VII. Conclusion

In this paper, we benchmark deep learning methods on the Kvasir-SEG dataset. We conducted thorough and extensive experiments for polyp detection, localisation, and segmentation tasks and shown how different algorithms performs on variable polyp sizes and image resolutions. The proposed ColonSegNet detected and localised polyps at 180 frames per second. Similarly, ColonSegNet segmented polyps at the speed of 182.38 frames per second. The automatic polyp detection, localisation, and segmentation algorithms showed good performance, as evidenced by high average precision, IoU, and FPS for the detection algorithm and DSC, IoU, precision, recall, F2-score, and FPS for the segmentation algorithm. While algorithms investigated in this paper show a clear strength to be used in clinical settings to help gastroenterologists for the polyp detection, localisation, and segmentation task, computational scientists can build upon these methods to further improve in terms of accuracy, speed and robustness.

Additionally, the qualitative results provide insight for failure cases. This gives an opportunity to address the challenges present in the Kvasir-SEG dataset. Moreover, we have provided experimental results using well-established performance metrics along with the dataset for a fair comparison of the approaches. We believe that further data augmentation, fine tuning, and more advanced methods can improve the results. Additionally, incorporating artifacts [73] (e.g., saturation, specularity, bubbles, and contrast) issues can help improve the performance of polyp detection, localisation, and segmentation. In the future, research should be more focused on designing even better algorithms for detection, localisation, and segmentation tasks, and models should be build taking the number of parameters into consideration as required by most clinical systems.
